# Sycotic Dysmenorrhœa

**Published:** 1893-02

**Authors:** George H. Clark

**Affiliations:** Germantown, Pa.


					﻿Third Day—Evening Session.
Thursday, June 23d, 1892, 8 p. m.
SYCOTIC DYSMENORRHGEA.
George H. Clark, M. D., Germantown, Pa.
As an illustration of the baneful effects of the syeotic poison,
the following case, one among several that have come under
notice in the past several years, will serve to give emphasis to
what has been written before on this subject.
Five years ago, a young woman, aet. seventeen years, came for
treatment for painful menses especially.
The previous history, as told by her mother, was that she had
always seemed to lack strength. Up to her fifth year she had
appeared well, but from that time she always complained of
being tired from the least exertion. She would awake in the
morning feeling worn out. A loose cough, without expectora-
tion, was the only other noticeable symptom. In appearance
she seemed perfectly well. She attended school regularly, and
had no other symptoms until she arrived at puberty, when the
lassitude became more prominent, particularly before and after
the menses.
The menses first appeared during her fifteenth year, and for a
few months there was little or no trouble, except the prostration.
Then she began to have more or less pain for the first two days
of the period, with weakness in the uterine region, and a feeling
as though the pelvic organs would fall out.
This continued, and after a few months more, with the pain
during menses, there were slight convulsions, which increased
from month to month until she came under my notice.
At each menstrual period there would precede the flow, for a
few hours, much pain in the uterine and ovarian regions, which
would disappear about four hours after the flow came on.
Remedies were given which gave much ease at the next period,
and then I would hear nothing from her.
At last there arose, in connection with the pain at each period,
symptoms that indicated more than simple dysmenorrhoea. The
menses would be preceded several hours by terrible convulsions,
which would continue until the flow appeared. There were
jerking of the head from side to side, opisthotonus, and forcible
flexion of the fingers—in other words, tonic spasms.
Considering these symptoms in connection with the marked
debility, I asked her father how long it had been since he had
gonorrhoea. He was surprised at my question, but acknowledged
that before marriage he had had two attacks. I also told him
he had been treated by injections, which he said was correct.
There was now no doubt of the nature of the difficulty. I in-
formed her mother that it would require some years toumake her
well, but I could promise she would be better in a few months,
if she would follow my advice. This they promised to do. She
began to improve at once.
The period following the remedy given for the convulsions,
.the pain, the debility, and the other symptoms, was much easier
than many of the preceding, and she continued to come for treat-
ment for about three months. Then I saw nothing more of her
for several months, when she re-appeared with the statement
that she thought she was cured, but that now her old symptoms
had returned.
She again improved under treatment, and again failed to ap-
pear for several months. At last her father came one day in
great haste and said : “ Can you do nothing for my daughter ?
I have just left her, and I have never seen such suffering. She
had an awful convulsion, which lasted two hours, and I am
anxious to know whether she can be helped.”
He was told that help had been given, and it was prom-
ised to be more permanent, provided my advice was followed.
That was to continue treatment for two years. The improve-
ment following the renewed treatment was as marked as before,,
and she has continued to come until the present.
In February last the menstrual period was delayed, and,
without consulting me, she took from a small vial which had
been given her two years ago, several doses in quick succes-
sion. Then followed symptoms from which she had been free
for several months.
Her mother wrote : “ My daughter had a spasm while sitting
up in bed at 11.30 o’clock. The flow appeared at 9 o’clock.
The pain across the abdomen was not so severe as formerly,
and she said she felt as though her back would break. The
spasms and vomiting continued until 4 o’clock. The spasms
were very severe; she gritted her teeth until I was afraid she
would break them off; clenched her hands, and twisted her
arms completely around ; her head was drawn back, and every
part of her body affected. She was much exhausted and then
fell into sleep. She had a wakeful night, and complains now
of her head feeling as though spikes had been driven through it,
and is passionate and irritable, and has spells of crying.”
The remedy she had taken without advice, and which had
been formerly given one dose each day two days before the time
for the menses, was Cuprum-acet.451".
A reference to its pathogenesis will show that it produces such
symptoms as she had.
The symptoms for which it had been given had disappeared,
and did not return until the remedy was taken when not neces-
sary. Stramonium was given as an antidote. After this I
went over her symptoms again, and the following were noted :
Great despondency; feels like crying all the time, but has no
cause for it. Is afraid to be alone, particularly at night. Fear
of going into a crowded place; does not want to go to church.
Constant anxiety. Dislikes to write a note, for fear she cannot
write correctly. Wishes to see none but members of the family.
Cannot concentrate her thoughts; thinks there is no use in read-
ing, for she cannot remember anything.
Pain and soreness in region of left ovary. Is better able to
walk without fatigue, but is still averse to doing anything.
Loose, rattling cough, worse on rising in the morning. Later
her mother informs me that at times she seems quite bright and
cheerful, but is mostly lamenting her condition, and thinks it a
hardship that she should be an invalid. She complains of hav-
ing a weak, nervous feeling constantly, is easily startled, but is
not now afraid to go about alone.
Medorrhinumdmm, is the remedy that has been acting since
she recovered from the effects of the Cuprum taken in Feb-
ruary.
Discussion.
Dr. Farley—I should like to hear how the Doctor decided
that the case was sycotic.
Dr. Clark—I decided after I learned the father had sycosis.
I had had several cases precisely the same as this, which were
sycotic, but I did not decide in this case positively until I had
asked the father. I knew her father very well; he had been
an invalid for forty years ; he had been Dr. Hering’s patient.
In walking he appeared to suffer from loss of the ligaments of
the head of the femur. Once when his daughter was very sick,
Dr. Lippe was called in consultation, then Dr. Fellger. Fellger
said there was loss of the capsular ligament, and also the liga-
mentum teres; and also that the father must have had some
venereal disease. I then concluded that it must be sycotio
dysmenotrhoea in the daughter. Since then I have had several
cases. As I said yesterday, Medorrhinum was a very disap-
pointing remedy to me. I have prescribed it in cases where it
was certainly indicated, with temporary relief only.
Dr. Tompkins—I should like to know whether this group of
mental symptoms is to be held as characteristic of sycosis. One
of our members gave me that impression. While Dr. Clark was
reading his paper I could almost have sworn he was reading
from notes of a case of my own, as far as the mental symptoms
go. Sepia was the sufficient remedy for the constipation, the
headache, and the mental symptoms.
Dr. Farley—Has that fishy odor been observed as particularly
characteristic of Medorrhinum ?
Dr. Baylies—Is that not characteristic of Sanicula and of an-
other drug, which I cannot recall this minute? (Tellurium.)
Dr. Clark—The mental symptoms are the most important
symptoms of all. Granvogel says : “No matter what the
pathological condition may be, there will always be these mental
symptoms in sycosis, and the morbid discharge from the mucous
membrane will be greenish.” His work is out of print, but I
presume it can be obtained.
Dr. Tompkins—It is to be hoped that not even so excellent a
remedy as Medorrhinum can lead us into the error of prescrib-
ing for a sycosis rather than for our patient. We must, I sup-
pose, individualize our cases of sycosis as carefully as any other.
CLINICAL CASES.
B. Fincke, M.D., Brooklyn, N. Y.
1.	Mrs. D. June 26th, 1859. 3 p. m.—Chill last night at
11 p. M. with pains in all the bones and slight thirst, lasting one
hour and a half; then fever, lasting till morning with perspira-
tion. Vomiting to-day. Pain in passing water. Constipation,
for which she took Castor Oil with immediate effect; also gin
and water without effect. 1^. Pulsatilla-nig. 5m (F.) three
pellets. Cured.
2,	Mrs. D. June 11th, 1864.—Felon on the right thumb for
one week. Cut this morning in several places, looking fright-
ful, suppurating, with violent sticking pains all along the arm.
The physician says she will lose her bone. The cutting made
everything worse. T^. Silicea 40m (F.); a few pellets in half a
tumbler of water; a teaspoonful every three hours. Seven powders.
June 15th.—The pains had subsided the second day. A large
red blister formed at the back of the thumb below the nail; the
thumb was in good shape and looked like healing. The woman
coming five miles distance was astonished at the result and got
well without anything else than the internal medication.
3.	Mrs. D. Forty years; pregnant. February 28th, 1867.
Echo of her own words, and of her own breathing; buzzing in
her right ear after 4 p. M. ft. Causticum cm (F.). Had an
astringent taste and cured.
4.	Mrs. S. January 3d, 1865.	11.15 A. m.—Since a fort-
night toothache, slowly increasing till about five days ago, when
the pains became very violent. Tearing and stinging and burn-
ing pains in the lower left three molars, later going into ears and
forehead; incessant; ameliorated by cold water held in the
mouth, and by external warmth. Tearing in the left leg below
the knee downward. Sensation between the shoulders as of ice.
■Chilliness, thirst. Three days ago the lower jaw began to swell.
Took yesterday Antimon, crud. without effect. She went to a
dentist to have one of the affected molars drawn, which felt three
times larger and had a flat cavity just above the gum, but the den-
tist refused because it would make her much worse. The woman
declared afterward that she had a good deal of toothache in her
life, but nothing like this before. She heated a knitting needle
red hot and burned the tooth, but the help it afforded did not
last long. Quarter past eleven o’clock A. M. I|«. Silicea cm (F.)
two pellets.
January 6th.—The toothache was better immediately and de-
creased gradually. The tooth feels a little larger yet, but is not
elongated. Pains almost all gone. Throbbing in the lower
jaw in the bone below. The day when she took the medicine,
between 3 and 4 P. m., violent tearing in the left cheek and
left ear, which felt as if ulcerated, and did not bear the least touch.
Yesterday terrible tearing pain in the left arm, mostly upper
arm, with numbness of the fingers. After this ceased it was as
if fire was running through with burning of the skin. Several
times a stitch below the right short ribs going half-way through
the body inward on a place where it was so painful yesterday,
as from ulceration, that she could not bear touching it. Yester-
day sensation in the splenic .region inside like ice, then turning
into violent heat for half an hour. She wakes up about 1 A. M.
and cannot sleep any more. She took nothing else and was
well.
5.	Mrs. S. May 23d, 1865.—Whilst washing, standing on
cold stones, on the third day of menstruation, this stopped,and,
suddenly, bearing down of the womb came on with terrible hot
cutting pains in the hypogastrium, more in the right side, going
down the thigh like wild labor pains and around the navel.
Half-past nine P. M. 1^. Pulsatilla-nig. 5m (F.).
After half an hour better. The pains went back very much
as they came like in emptying a bottle, then heat and burning
in the stomach, going up to oesophagus and out of the mouth..
The places of pain are now sore as from a wound.
June 10th.—Menses did not return-, but she was well.
6.	George L-------, merchant, August 9th, 1864, lived in
Gowanus last May, got chills and fever, and was treated with
Quinine. Chill came on at 10 A. M. and lasted till noon, fol-
lowed by perspiration, with thirst through all the stages. It
came on four or five times as tertiary, then it stopped. Took
Quinine, seven grains at once, then five grains three times
every day for eight days. Then the fever was gone.
But since then he gets the fever sometimes in the forenoon.
Several days preceding it, tired pains in sacrum and lower ex-
tremities. Yesterday tired, languid, white fingers. Chill for
one hour, shaking, some headache, nausea, then heat for fifteen
minutes, then sweat for three hours. Urine dark with dark red
sediment. Stool normal. After having had the fever a second
time, swollen feet and eruption around lips and nose for eight
days. Some yellowness of conjunctiva. Tongue rose-colored
with enlarged papillae, but when he has the fever the tongue is
coated whitish-yellow. Sometimes gets an attack in one, two
or five weeks, and he takes Quinine between times. Sleeps well,
has an inborn aversion to wine, beer or other spirituous bever-
ages, but loves coffee and tea passionately. Smokes a good deal.
Had an attack of cholera five years ago treated allopathically
by Opium. 9 A. m., I|«. Natrum-mur. 5m (F.). Several years
after I learned from a patient sent‘by him that this dose cured
him right up.
7.	F. M-----, a music teacher, short, thick-set, March 5th,
1874.—Had given a lesson in a heated room, gets a cold perspira-
tion, nausea, a dreadful colic, the cold sweat running in streams
all over his body, and he thinks he must die. 4 P. M., R.
Arsenicum-a. 90m (F.). In ten minutes it was all gone and he
was well.
8.	Dr. B-----, July 22d, 1867.—Sciatica in left lower limb,
worse on coughing, laughing, sneezing, going to stool, straight-
ening the limb, stepping lower than imagined. Rheumatism
in the small of back preceded it. The pain goes from the fora-
men sciaticum down to the foot, which is numb. Suffers since
June 28th. R. Tellur-met. 90m (F.). May 24th, 1874.—
Helped him and since then he cures all the sciaticas of his
patients with Tellur. 90m. So he told me to-day.
9.	E. K.-----, nineteen years, dark, florid complexion, tall
and thin. October 12th, 1866, quarter of three p. m., was brought
to mv office by the late apothecary, Mr. Smith, bleeding from the
nose profusely. I gave him at once a dose of Aeon, cm (F.), which
stopped the bleeding in a few minutes while in the office, and he
did not bleed for two months after, when a little blood came
from the right nostril.
This case was a fearful one. He was taken in Nassau Street,
New York, bleeding all the time. When arriving at Smith’s
store the blood was streaming from both nostrils and filled a
basin full. Mr. Smith held a piece of ice six inches square on
the back of the neck, but it was not of the least avail. The
bleeding continued all the way and here, till Aeon, cm stopped it.
That boy must have had a greater amount of blood in him than
common. And yet he did not show much weakness, for he
walked home again in a short while. He had valvular disease
of the heart for which I treated him. For half a year he did
not come near me because he felt so well. But then he struck
his nose and bled for five minutes, when it stopped by itself.
Later on, when I treated him for his heart disease, he bled again
for an hour after over-exertion in warm weather, and that was
the last I saw of him. He went from me to Dr. Boskowitz,
and from him to an allopathic physician, under whose care
he died a couple pf years later, having got married in the mean-
time.
10.	Mr. G., forty-five years, slight frame, merchant, July
22d, 1867. Since July 4th, from ice-cream and new potatoes,
sinking in stomach. Bad taste in mouth. Heavy weight two
and a half inches below the liver in the axillary line—“the
hand can grasp it”—Dr. Willard Parker who examined him
said it was not the liver—steady dull though not constant pain
eased by hot cloths. Appetite tolerable. Qualmish, nauseous..
Fried oysters, raw chestnuts disagree with him. Offensive
breath. Sleeps badly. Confused dreams. When waking up,
swelling of penis. Tongue slightly coated. Nasal catarrh,
matter forming in the nose. No sense of smell. From any
little cause, cold, or indigestion all the symptoms re-appear.
Hands dry. Feet cold. Want of vital heat. Active exercise
weakens him too much. Low-spirited, feels like crying. Nux-
vom. 50m (F.).
Got well shortly and ever since remained well.
11.	Mrs. W., fifty-odd years, February 15th, 1872.—Had se-
vere pain in the pelvic region which made it difficult for her to
walk, and rubbed on a mixture of Aconite tincture and Chlo-
roform, though she ought to have known better, because on a
former occasiou she had used Chloroform and did not get over
its effects for three to four years.
Now she is in an agony of pain, shrieking. Dr. R., a former
homoeopath, had diagnosed it a severe attack of hysteria, and
gave Asafoetida. He said she could have smeared Aconite
tincture all over her body and it would not have affected her in
the least, which the old gentleman doubted, for he had seen a
little Aconite tincture put over the eyelid, making the person
immediately blind. 1^. Aconit.-nap. 3cm, in water, a teaspoon-
ful every half-hour.
She was easier after an hour, and slept through the afternoon.
12.	Thomas H., an Irish nursling, six months. August
29th, 1854.—Suffers for the last two months from vomiting and
diarrhoea, since eleven days much worse. In this hot and
dry weather patient was emaciated to a skeleton. Hands
and feet cold. Incessant vomiting of greenish water and the
milk taken. Yellow watery diarrhoea. Abdomen hard. Shrill
crying. Convulsive movements of hands and feet. Sleep with
half-open eyes. Boring the head into the pillow, hands reach-
ing toward the back of the head. The child had convulsions
two months ago, and has no tooth yet.
Verat-a. 2400 (F.), one pellet at once to the child’s tongue.
Verat-a. 12 (F.), for the mother to be taken in half a tum-
bler of water, one teaspoonful every three hours.
August 31st.—The child is reported to be well. It did not
get sick till next summer, when a slight attack was easily dis-
posed of.

				

